# Medical Applications of Microwave Imaging

**DOI:** 10.1155/2014/147016

**Published:** 2014-10-14

**Authors:** Zhao Wang, Eng Gee Lim, Yujun Tang, Mark Leach

**Affiliations:** Xi'an Jiaotong-Liverpool University, Suzhou 215123, China

## Abstract

Ultrawide band (UWB) microwave imaging is a promising method for the detection of early stage breast cancer, based on the large contrast in electrical parameters between malignant tumour tissue and the surrounding normal breast-tissue. In this paper, the detection and imaging of a malignant tumour are performed through a tomographic based microwave system and signal processing. Simulations of the proposed system are performed and postimage processing is presented. Signal processing involves the extraction of tumour information from background information and then image reconstruction through the confocal method delay-and-sum algorithms. Ultimately, the revision of time-delay and the superposition of more tumour signals are applied to improve accuracy.

## 1. Introduction

Breast cancer has the highest mortality rate due to malignant tumour disease for women worldwide. As data published by the National Breast Cancer Coalition (NBCC) shows, there are 3 million women living with breast cancer in the United States alone [1]. The problem of avoiding the menace of breast cancer is receiving increasing attention. Experts suggest that the best defence against breast cancer is through early detection and treatment.

One of the most popular imaging methods in current breast cancer detection is X-ray mammography. However, sensitivity and specificity limitations result in a relatively high false positive rate (between 4% and 34%) [2] as well as a high false negative rate (up to 70%) [3]. In addition to this inaccuracy, X-rays are not suited to iterative use, especially for women under 40 years old due to their potentially harmful nature. Another technology, magnetic resonance imaging (MRI), has a higher sensitivity than X-rays in detecting breast cancer which means that MRI is far more susceptible to the detection of suspicious tissues that are not tumours and which results in a high rate of false positives [4]. Recently, ultrawideband (UWB) microwave imaging has been widely researched for early breast cancer detection for its safety, low cost, and high contrast [5].

Microwave images for medical applications are maps of the electrical property distributions in the body which have been paid close attention to for several years. It is defined as observing the internal structure of an object by means of electromagnetic fields at microwave frequencies (300 MHz–30 GHz). Breast cancer detection with microwave imaging is based on the contrast in electrical properties of cancerous tissues compared to normal tissues. The imaging process is anticipated to be rapid, sensitive (detect most tumours in the breast), and specific (detect only cancerous tumours) as well as offering patients a more comfortable and safer examination procedure.

**Figure 1:**
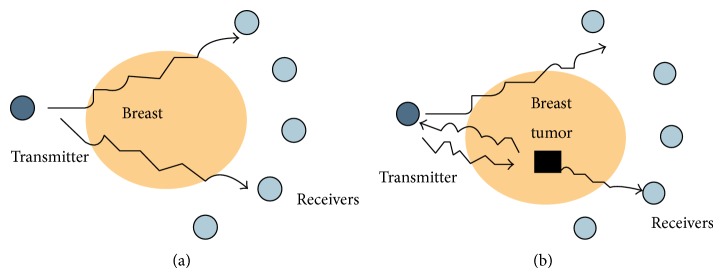
Signal transmission of breast cancer detection.

The microwave energy travels through the breast from a transmitter and is detected at receivers located on the other side of the breast as shown in Figure 1. Simultaneously, reflections may be recorded at the transmitter. Microwaves traveling through the tumour experience a change in material dielectric property which leads to scattering of the incident wave. This scattering alters the energy detected at the receivers and the transmitter also shown in Figure 1. Finally, images are formed from the information of detected energies. This implies at least two approaches for creating microwave images which are tomography and radar based technology.

In this paper, the detection of the malignant tumour is performed through the application of feasible signal processing techniques. A 3D model of a breast containing a malignant tumour, characterised by known electrical properties of tissues, has been developed in an appropriate electromagnetic simulation tool so that the fields formed by the scattering of a UWB microwave signal in the breast can be found. Once the scattered electromagnetic field distribution is obtained from the simulation, signal processing techniques involving the confocal method by delay and sum algorithms can be used to reconstruct the breast image and identify the waves relating to the tumour.

## 2. Microwave Imaging Model

Tomography is a transmission-reflection imaging method using numerous antennas surrounding an object being imaged, in this case the breast. The shape of the target and dimensional distribution of the permittivity are acquired from the incident (transmitted) and scattered (received) fields. On the other hand, radar based technology only uses reflections from the object. A system generates a microwave pulse from a transmitter and receives it in a uniform location with uniform antenna, as illustrated in Figure 1. Radar systems may use modulated harmonic microwave signals instead of a pulse or use an alternative location for the receiver from that of the transmitter [6–8].

Distinction of breast-tissue using the pulsed microwave confocal approach is based upon two primary physical properties. The first is that the high-water content of malignant tumours causes them to have significantly larger microwave scattering cross sections than the low-water content normal fatty breast tissues [9]. The vascularization of malignant tumours also further enhances the scattering cross section. Secondly, the low-water content of fatty breast-tissue means that the absorption coefficient is low at microwave frequencies and therefore is suited for wide-bandwidth backscattered returns and measurement using broad-aperture confocal-imaging techniques. It is found that the dielectric properties of normal breast tissues are similar to fat, while the properties of malignant breast tumours resemble muscle. According to data measured by many sources [10], the dielectric properties of normal breast tissues vary in an approximate 10% range around *ε*_*r*_ = 9 and *σ* = 0.4 S/m, whereas, for malignant tumours, *ε*_*r*_ = 50 and *σ* = 4 S/m.

**Figure 2:**
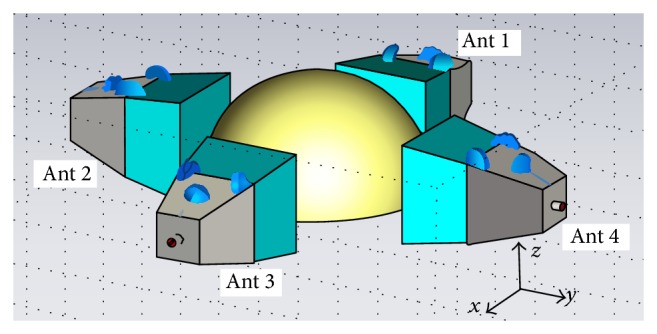
Simulated breast model constructed in simulation tool.

The simple breast model is constructed in CST microwave studio as presented in Figure 2. It is a hemisphere with the sphere centred at (0,0, 0) and a radius of 50 mm containing just breast tissue, with no skin layer (which has a different set of electrical properties) or glandular organ and vasculature structures for ease of modelling and imaging. As shown in Figure 2, four horn antennas encircling the breast at equal heights are used to transmit and receive the microwave signals. The aperture centre of antenna 1 is located at −55,0, 10, aperture centre of antenna 2 is located at 0, −55,10, aperture centre of antenna 3 is located at 55,0, 10, and aperture centre of antenna 4 is located at 0,55,10. The background material should be set as a coupling medium with *ε*_*r*_ = 9 to ensure electrical matching between antenna and internal breast.

The time-delay *t* for the propagation of the microwave signal in a given pair of transmitting/receiving antenna is calculated based on the antenna's position, position of the focal point *r* = (*x*, *y*, *z*), and an estimate of average wave propagation speed. During focusing, the focal point is moved from one position to another within the breast to create spatial beam-forming. All time-shifted responses are coherently summed and integrated at each focal point. Integration is performed on the windowed signal, and the length of the integration window is chosen according to the system bandwidth. Due to the antenna effects and dispersion, the integration window we utilize following coherent summation is 50 percent longer than the duration of the synthetic input pulse. The main advantage of the delay-and-sum algorithm is its simplicity, robustness, and short computation time.

Fundamentally, the energy at the focal point in the breast can be calculated by

(1) $$\begin{array}{c} E\left(x,y,z\right)=\overset{\tau }{\underset{0}{\int }}{\left(\overset{M}{\underset{1}{\sum }}y\left(t-T\left(x,y,z\right)\right)\right)}^{2}dt, \end{array}$$

where *M* is the total amount of received signal energy, *y* is simulated received energy signal, *T* is the time-delay of each focal point, and *τ* is integration range.

Supposing that the focal point is at (*x*, *y*, *z*) with a distance *d* to antenna *i*, the time-delay can be expressed as

(2) di=(x−xi)2+(y−yi)2+(z−zi)2v,

where *v* is the spread velocity in the breast-tissue which can be also calculated as

(3) ν=με2[1+(σωε)2+1]−0.5=92[1+(0.49×107π)2+1]−0.5=0.99×108 m/s.

There is a finite distance from the port location to the centre of the antenna aperture surface position; hence the time-delay should include both the value calculated by the equation above and the port to antenna aperture surface time-delay *dt*.

## 3. Simulation

**Figure 3:**
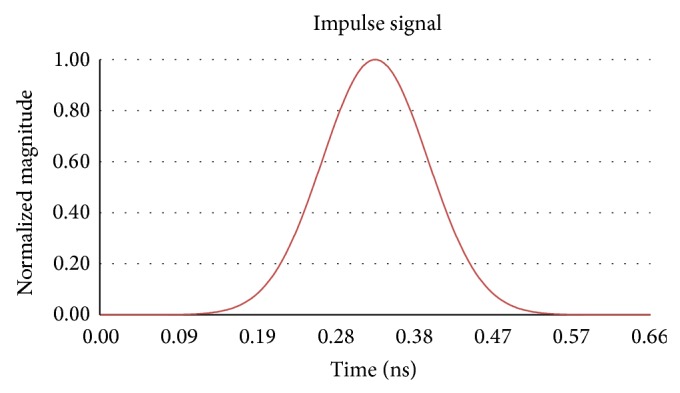
Transmitted Gaussian impulse signal.

**Figure 4:**
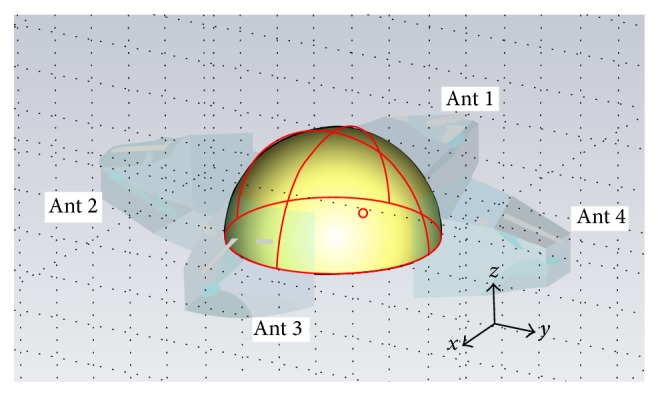
Breast model with tumour constructed in CST.

In the simulation, antenna 1 is selected as the transmitter and antennas 2 to 4 are utilised as receivers. The transmitted signal is a Gaussian pulse with a bandwidth of 5 GHz and its shape in the time domain is shown in Figure 3. The model is simulated by running the transition solver in Computer Simulation Technology (CST) Microwave Studio, set for an accuracy of −50 dB. The model records the responses at the ports of antennas 2–4 for the transmitted pulse from antenna 1. These responses represent the state of the breast with no tumour present. The model is then modified to include a spherical tumour with a 2 mm radius within the breast-tissue centred at a location of −10,10,10 as shown in Figure 4. The model was resimulated and the results were recorded.

**Figure 5:**
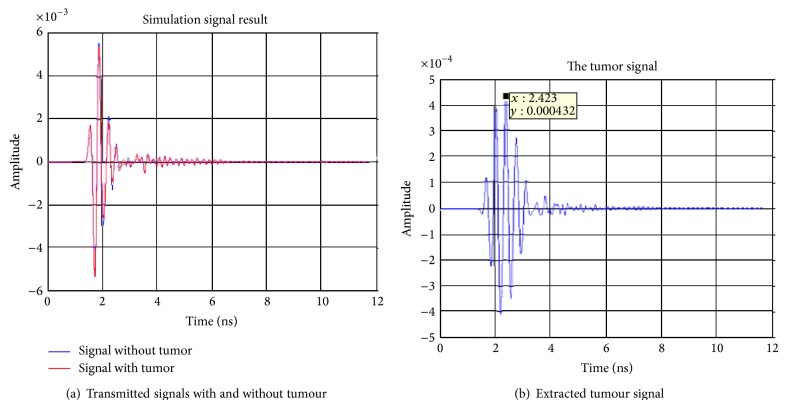
Simulation signal results transmitting from antenna 1 and receiving at antenna 2.

**Figure 6:**
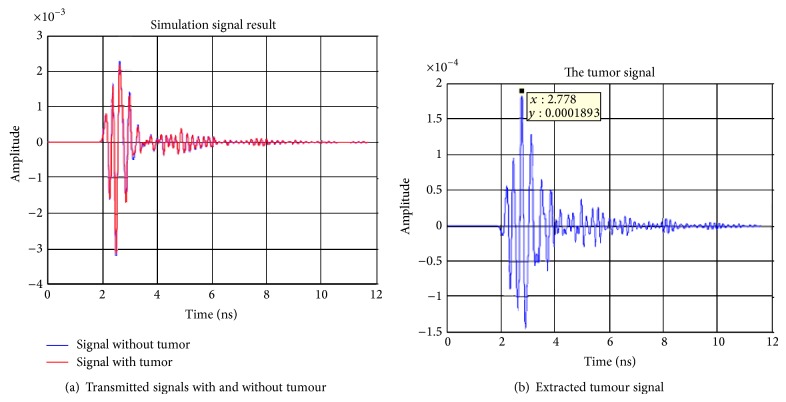
Simulation signal results transmitting from antenna 1 to antenna 3.

**Figure 7:**
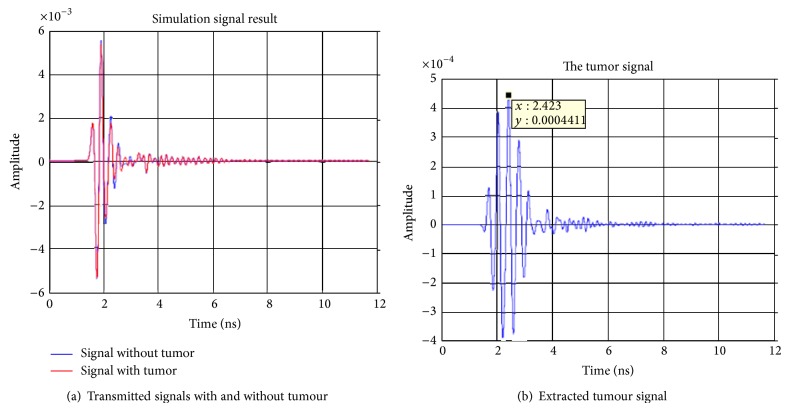
Simulation signal results transmitting from antenna 1 to antenna 4.

The first signal processing step is to deal with the extraction of the tumour response from the raw measured data. This can be achieved through a simple subtraction of the data obtained for the breast without the tumour from that of the breast with the tumour. The raw and subtracted data obtained for receiving antennas 2–4 are shown in Figures 5, 6, and 7. From these figures, it can be observed that the maximum voltages (amplitude) appear at a time of 2.423 ns, 2.778 ns, and 2.423 ns in antennas 2, 3, and, 4 respectively. The locations of the maximum received signal strength at each receiver imply the length of the time delay for the signal to travel from the transmitter through the imaging plane to the receiver.

**Figure 8:**
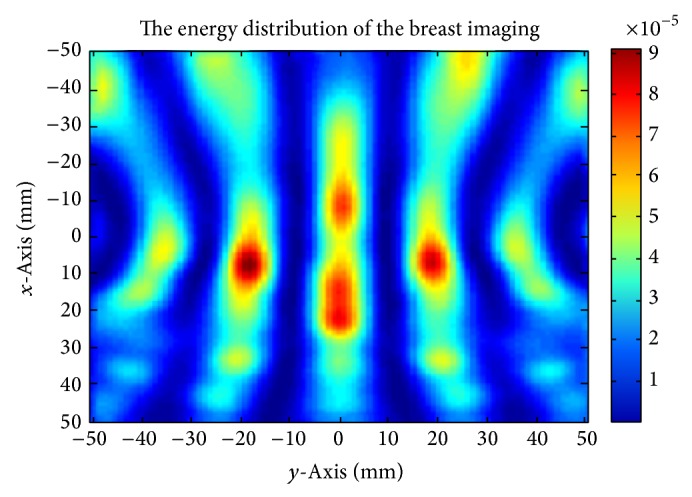
Reconstruction of the breast image.

The final planar view of the focused image can be obtained through the application of (1) and is shown in Figure 8. In this image of the *z* = 10 plane, the vertical axis represents the *x* direction and the horizontal axis represents the *y* direction. In Figure 4 strong energy locations can be identified, the strongest of which is located at 10, −20. In comparison to the actual location of the tumour at −10,10 the calculated data represents a large deviation from the model.

The delay-and-sum algorithm requires precise timing to be applied to the relative signals. Through further consideration of the respective delay time to be applied to each signal, despite the implied time delays from the responses in Figures 5–7, manual calculation of the time-delay based on (2) and (3) leads to delays of 1.2901 ns, 1.6481 ns, and 1.4917 ns for each antenna, respectively.

**Figure 9:**
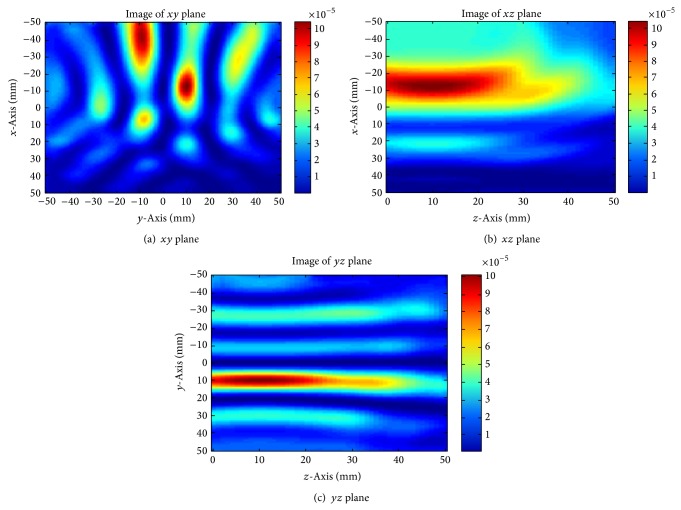
Breast images of changing precise time delay.

On recalculation of (1) with these new time delays, a focused image is obtained.  Figure 9 shows cuts through the *xy* plane, *xz* plane, and *yz* plane that intersect with the tumour location. The strongest energy points are located at −10,10 in the *xy* plane, −10,10 in the *xz* plane, and 10,10 in the *yz* plane. This indicates that the tumour is located at −10,10,10 inside the breast model, coinciding with the location used in the model. This result verifies that delay-and-sum algorithm is applicable in breast cancer detection, providing an adequately precise time-delay is obtained to impart a suitable shift on the received signals. More investigations are also required into the equalization of tissue losses as well as performing equalization of the radial spread of the spherical wavefront to guarantee that all received pulses are of the same shape and amplitude and perfectly time-aligned. This will eliminate more errors, thus improving image quality.

**Figure 10:**
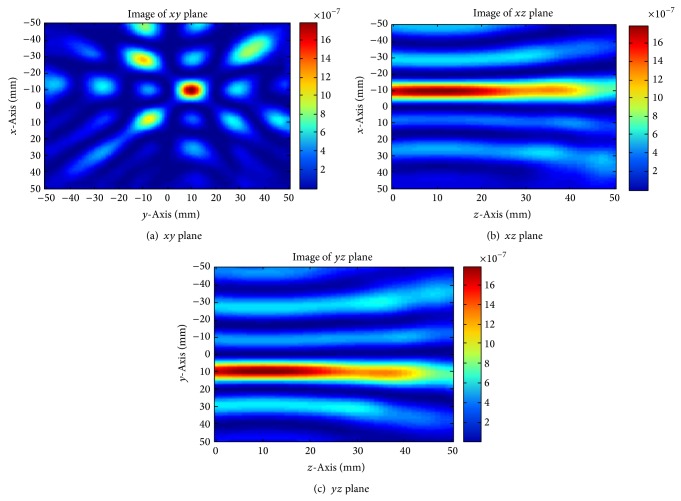
Breast image through addition of tumour signals.

The signal processing applied in the simulation (as shown in Figure 9) used only 3 received tumour signals as a reference. For the purpose of increasing energy at the focal point of the tumour, more tumour signals can be added into the delay-and-sum algorithm. This can be achieved in a straightforward manner by using each of the 4 antennas in turn as the transmitter and the other 3 as receivers. A total of 12 tumour signals can be obtained in this manner. The reconstructed images are presented in Figure 10.

From Figure 10(a) it is clear that the tumour location has become better defined in comparison to that obtained in Figure 9(a), while the images in the *xz* and *yz* plane are still not markedly improved in tumour shape definition. This is due to the antenna location. In this simulation, the antennas are located encircling the breast on the *xy* plane which results in better definition in this plane. To obtain the same level of definition in the other planes, the receiving antennas need to be distributed around the plane of interest. This implies that using more antennas to surround the breast a high quality 3D tumour image could be obtained.

## 4. Conclusion

In this project, the delay-and-sum algorithm has been shown to provide appropriate levels of accuracy when used in microwave imaging, in particular for the application of breast cancer detection. It should be noted that its use requires a highly accurate calculation of the time-delay between transmitter and focal point to receiver. In addition, it has been shown that the use of more signals in the image reconstruction process can dramatically improve image definition. The time-delay between the antenna port and the antenna aperture surface remains an important factor in image reconstruction and will be investigated as further work. A solution to this problem that does not involve manual calculation based on known tumour position needs to be found. Moreover, loss equalization prior to signal processing and 3D imaging through the addition of more antennas on different planes should be considered.
